# Aldosterone-Induced Transformation of Vascular Smooth Muscle Cells into Macrophage-like Cells Participates in Inflammatory Vascular Lesions

**DOI:** 10.3390/ijms26073345

**Published:** 2025-04-03

**Authors:** Boya Zhang, Ziqian Liu, Yi Chang, Ruyan Lv, Haixia Guo, Panpan Qiang, Tatsuo Shimosawa, Qingyou Xu, Fan Yang

**Affiliations:** 1 Graduate School, Hebei University of Chinese Medicine, Shijiazhuang 050200, China; 2 Hebei Key Laboratory of Integrative Medicine on Liver-Kidney Patterns, Hebei University of Chinese Medicine, Shijiazhuang 050200, China; 3 Institute of Integrative Medicine, College of Integrative Medicine, Hebei University of Chinese Medicine, Shijiazhuang 050200, China; 4 Department of Clinical Laboratory, School of Medicine, International University of Health and Welfare, Narita 286-8686, Japan

**Keywords:** vascular smooth muscle cell, macrophage-like cell, aldosterone, mineralocorticoid receptor blocker, macrophage colony-stimulating factor

## Abstract

Vascular smooth muscle cells (VSMCs) are the most abundant cell type in blood vessels, participating in cardiovascular diseases in various ways, among which their transformation into macrophage-like cells has become a research hotspot. In this study, rats were infused with aldosterone for 12 weeks, and VSMCs stimulated with aldosterone in vitro were used to observe aortic injury and the role of VSMC transformation. Vascular changes were detected via small animal ultrasound and H&E staining. Moreover, immunohistochemistry, immunofluorescence, Western blot, and flow cytometry were used to verify that the transformation of VSMCs into macrophage-like cells is regulated by mineralocorticoid receptor (MR) activation and macrophage colony-stimulating factor (M-CSF) and its receptor. Rat vasculature and in vitro cellular experiments revealed that VSMCs transformed into macrophage-like cells and secreted inflammatory factors such as interleukin-1β (IL-1β) and monocyte chemoattractant protein-1 (MCP-1), thereby exacerbating inflammatory vascular lesions, which was inhibited by the MR antagonist esaxerenone. These results reveal that increased levels of aldosterone activate MR, leading to the secretion of M-CSF by VSMCs. This further promotes the transformation of VSMCs into macrophage-like cells, which participate in inflammatory vascular lesions. Therefore, inhibiting the formation of macrophage-like cells can effectively reduce inflammatory vascular lesions.

## 1. Introduction

Vascular injury is the pathological basis of cardiovascular disease (CVD). Pathological changes in blood vessels, such as intimal thickening and plaque formation, are typical manifestations of arterial injury and are common in hypertension and coronary heart disease (CHD) [1,2,3]. Vascular smooth muscle cells (VSMCs) are the most plentiful cell type in blood vessels and participate in vascular physiological functions and vascular diseases in different ways; in particular, VSMC phenotypic transformation plays an important role in various CVDs, such as atherosclerosis, aortic aneurysm, and vascular calcification [3,4,5,6].

Macrophages that result from VSMC transformation are called macrophage-like VSMCs, and the participation of macrophage-like VSMCs in the formation of vascular intimal plaques upon their transformation into foam cells is a hotspot of research on the pathogenesis of atherosclerosis [7,8,9]. The transformation of VSMCs into macrophages is regulated by a variety of mechanisms, among which lipid metabolism has been studied in depth [10,11,12,13]. Additionally, inflammation plays a central role in the occurrence and development of CVDs, such as CHD, hypertension, and chronic heart failure [14,15]. Inflammation prompts macrophages to engulf oxidized low-density lipoprotein (oxLDL) cholesterol and convert it into foam cells, which is a key process in atherosclerosis. Interestingly, not all macrophages that convert into foam cells are of myeloid origin, but approximately 30%~40% are derived from VSMCs [16]. Cells in the human aorta that coexpress α-smooth muscle actin (α-SMA) and CD68 were identified as early as 1997 [17].

Aldosterone, an important proinflammatory mediator, plays an important role in cardiovascular injury [18]. Moreover, mineralocorticoid receptor (MR) activation is a key pathophysiological change in CVD [19]; thus, MR antagonists (MRAs) have also been widely used to treat CVD [20]. However, further verification is needed to determine whether the protection offered to blood vessels by MRAs is related to the inhibition of the macrophage-like cell transformation of VSMCs.

In this study, we explored the role of aldosterone in the induction of the VSMC macrophage-like transformation in vivo and in vitro. Our data provide a basis for the mechanism by which aortic injury can be induced via the MR-mediated upregulation of macrophage colony-stimulating factor (M-CSF) during cell transformation and verify the antagonistic effects of esaxerenone.

## 2. Results

### 2.1. Aldosterone Induced Inflammatory Vascular Lesions

**Figure 1:**
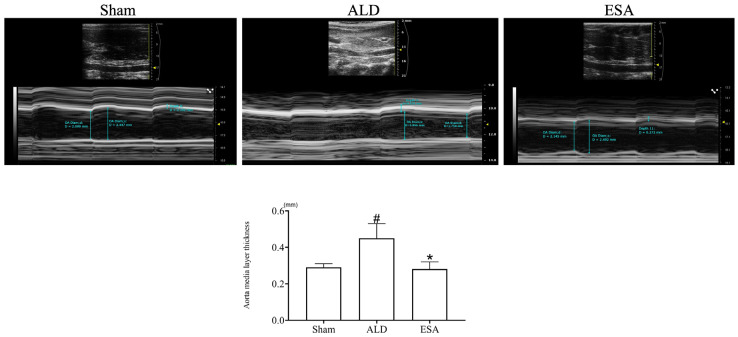
Intravascular ultrasound imaging of the aorta. ^#^ *p* < 0.05 compared with the Sham group, and * *p* < 0.05 compared with the ALD group. ALD: aldosterone stimulation; ESA: esaxerenone treatment.

To confirm the changes that occur in blood vessels after aldosterone infusion, we performed ultrasonography and found that compared with that in the Sham group, the aorta media layer in the ALD (aldosterone) group was significantly thicker, whereas the thickness in the ESA (esaxerenone) group was lower than that in the ALD group (Figure 1).

**Figure 2:**
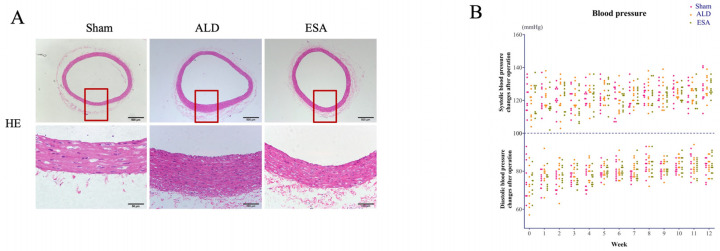
Aldosterone-induced inflammatory vascular lesions. (**A**) H&E-stained images for the evaluation of aldosterone-induced inflammatory vascular lesions. The part enclosed by the red box is zoomed in. Scale bars = 500 μm, 100 μm. (**B**) Postoperative blood pressure measurements in each group of rats. ALD: aldosterone stimulation; ESA: esaxerenone treatment.

We then examined the changes caused by aldosterone to the aorta via hematoxylin and eosin (H&E) staining. Compared with that in the Sham group, the aortic media layer in the ALD group was thicker, whereas esaxerenone alleviated the thickening of the aortic media layer (Figure 2A). Moreover, the blood pressure in each group was also monitored, and no significant changes were observed (Figure 2B).

### 2.2. Transformation of VSMCs and Detection of the Macrophage-like Cell Type

**Figure 3:**
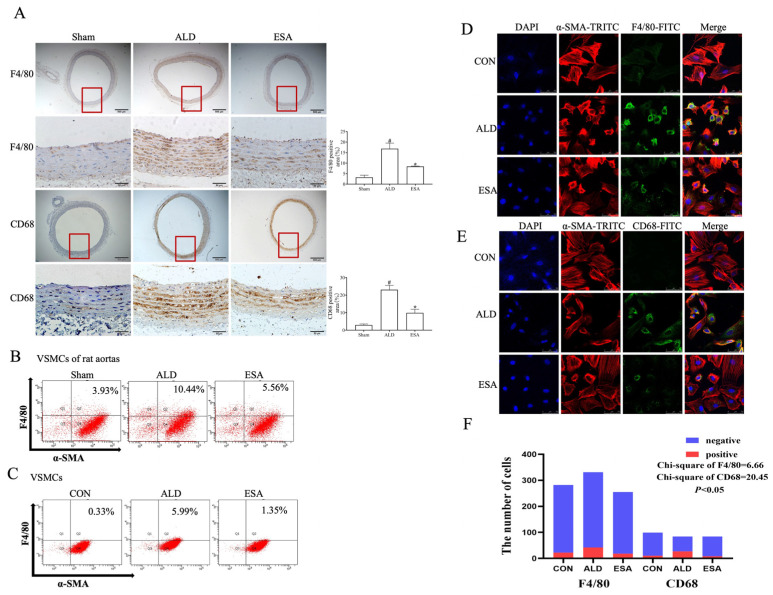
Esaxerenone inhibited ALD-induced vascular smooth muscle cell (VSMC) transformation. (**A**) Immunohistochemical staining of F4/80 and CD68 in the aortas of aldosterone-infused rats. The part enclosed by the red box is zoomed in. Scale bars = 500 μm, 50 μm. *n* = 3. ^#^ *p* < 0.05 compared with the Sham group, and * *p* < 0.05 compared with the ALD group. (**B**,**C**) Flow cytometry detection of F4/80 and α-SMA coexpression in VSMCs from the aortas of aldosterone-infused rats and VSMCs. *n* = 3. (**D**–**F**) Immunofluorescence staining of F4/80 and CD68 in aldosterone-stimulated VSMCs. Scale bars = 100 μm, 200 μm. *n* = 3. ALD: aldosterone stimulation; ESA: esaxerenone treatment; and α-SMA: α-smooth muscle actin.

To verify that VSMC-to-macrophage-like cell transformation occurs and determine its role in inflammatory arterial tissue lesions, we conducted both in vitro and in vivo experiments. We used immunohistochemical staining to examine the influence of aldosterone on F4/80 and CD68 expression in the arteries of rats. Compared with those in the Sham group, the expression of both F4/80 and CD68 in the aorta was greater in the ALD group, whereas the expression of both F4/80 and CD68 was lower in the ESA group than in the ALD group (Figure 3A). Additionally, flow cytometry was applied to detect F4/80 and α-SMA coexpression in the rat aortas and VSMCs. The proportion of α-SMA^+^F4/80^+^ cells significantly increased upon aldosterone stimulation compared with the Sham group, whereas the proportion of α-SMA^+^F4/80^+^ cells decreased after esaxerenone treatment compared with the ALD group (Figure 3B,C). Then, we performed immunofluorescence staining on VSMCs to confirm the effect of aldosterone on the VSMC-to-macrophage-like cell transformation and found that aldosterone stimulation increased the proportion of α-SMA^+^F4/80^+^ or α-SMA^+^CD68^+^ VSMCs, whereas esaxerenone decreased the expression of these markers (Figure 3D–F). The dynamic *Z*-axis variations observed in F4/80/α-SMA immunofluorescence co-staining enhanced visualization of the global morphological changes in the VSMCs (Figure S1). Taken together, these results suggest that VSMCs tend to transform into macrophage-like cells upon aldosterone stimulation and that esaxerenone inhibits this transformation.

**Figure 4:**
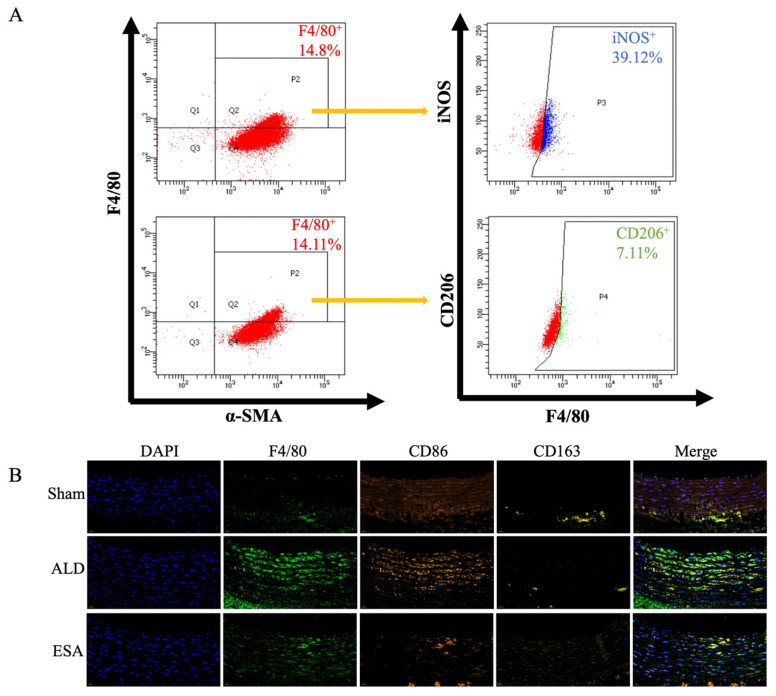
Effects of aldosterone on VSMC transformation indicators. (**A**) Flow cytometry was used to analyze the types of macrophage-like cells. *n* = 3. (**B**) Expression of macrophage subtype markers detected by four-color fluorescence staining: F4/80 is shown in green, CD86 is shown in orange, CD163 is shown in yellow, and DAPI is shown in blue. Scale bars = 20 μm. *n* = 3. ALD: aldosterone stimulation; ESA: esaxerenone treatment.

To further elucidate the subtypes of macrophage-like cells transformed from the VSMCs, we used flow cytometry to analyze the aldosterone-stimulated VSMCs. The macrophage-like cells were characterized by dual positive expression for F4/80 and α-SMA, after which the expression of iNOS (an M1 macrophage marker) and CD206 (an M2 macrophage marker) was detected in these cells. The proportion of M1 macrophages was 39.12%, whereas the proportion of M2 macrophages was 7.11% (Figure 4A). We validated these results via immunofluorescence staining and labeling with F4/80, CD86 (a marker of M1 macrophages), and CD163 (a marker of M2 macrophages), and compared with those in the Sham group, F4/80 coexpression with CD86 was greater than F4/80 coexpression with CD163 in the ALD group (Figure 4B). Furthermore, the VSMCs were treated with TGF-β, and the expression of α-SMA, F4/80, iNOS, and CD206 was subsequently measured to clarify the effects of aldosterone on cell subtypes. The results revealed that M1 macrophages were responsible for 10.95% of the macrophage-like cells, whereas M2 macrophages accounted for 23.58% (Figure S2). The above results confirmed that the macrophage-like cells resulting from the VSMC transformation upon aldosterone stimulation were mainly the M1 type.

**Figure 5:**
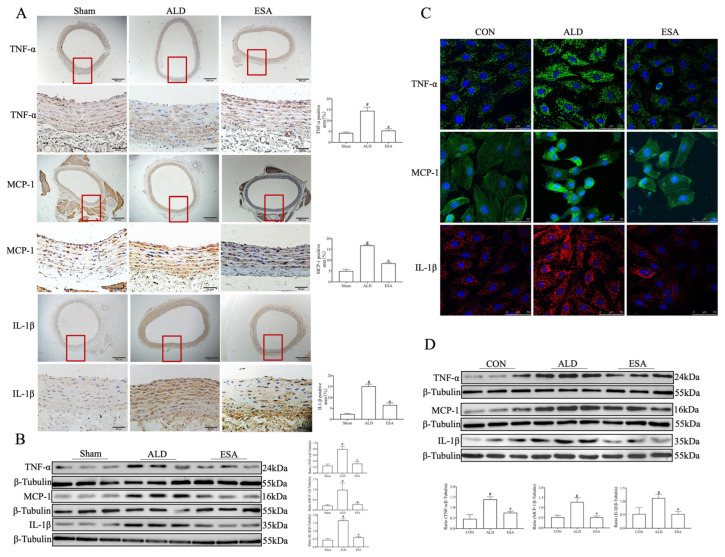
Effects of aldosterone on proinflammatory cytokines. (**A**) Immunohistochemical staining of TNF-α, MCP-1, and IL-1β in the aortas of aldosterone-infused rats. The part enclosed by the red box is zoomed in. Scale bars = 500 μm and 50 μm. *n* = 3. (**B**) Western blot detection of TNF-α, MCP-1, and IL-1β expression in the aorta. *n* = 6. (**C**) Immunofluorescence staining of TNF-α, MCP-1, and IL-1β in aldosterone-stimulated VSMCs. Scale bar = 50 μm. *n* = 3. (**D**) Western blot detection of TNF-α, MCP-1, and IL-1β expression in aldosterone-stimulated VSMCs. *n* = 6. ^#^ *p* < 0.05 compared with CON; * *p* < 0.05 compared with ALD. ALD: aldosterone stimulation; ESA: esaxerenone treatment; TNF-α: tumor necrosis factor-α; MCP-1: monocyte chemoattractant protein-1; and IL-1β: interleukin-1β.

We next detected proinflammatory cytokine markers by immunohistochemical staining and Western blot to determine whether inflammatory lesions had occurred in the aorta. The results showed that the expression of tumor necrosis factor-α (TNF-α), monocyte chemoattractant protein-1 (MCP-1), and interleukin-1β (IL-1β) in the ALD group was greater than that in the Sham group, whereas the levels of these indicators were lower after esaxerenone treatment (Figure 5A,B). Furthermore, immunofluorescence staining and Western blot analysis of TNF-α, MCP-1, and IL-1β expression in the VSMCs showed the same trend as described above (Figure 5C,D). These results suggest that aldosterone-induced inflammation may be involved in inflammatory aortic lesions in rats.

### 2.3. The MR/M-CSF Pathway-Mediated Transformation of VSMCs into Macrophage-like Cells Is Induced by Aldosterone

**Figure 6:**
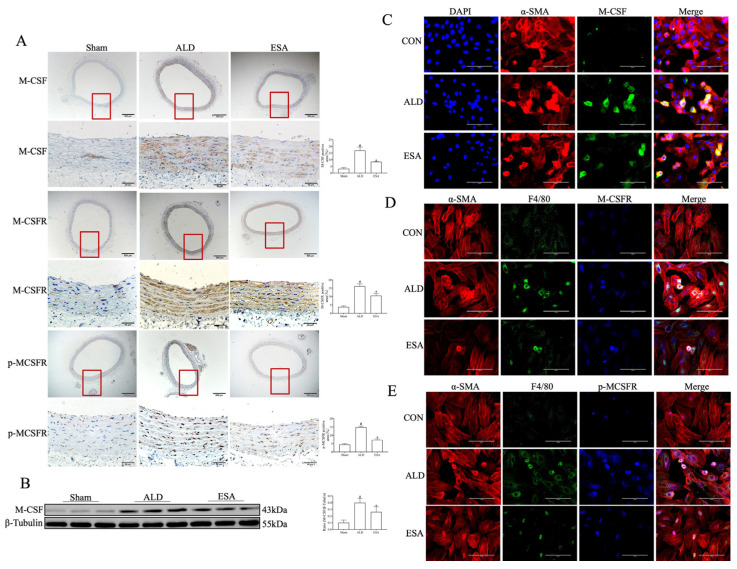
(**A**) Immunohistochemical staining of M-CSF, M-CSFR, and p-MCSFR in the aortas of aldosterone-infused rats. The part enclosed by the red box is zoomed in. Scale bars = 500 μm, 50 μm. *n* = 3. (**B**) Western blot was used to analyze the expression of M-CSF in rat aortas. *n* = 6. ^#^ *p* < 0.05 compared with the Sham group; * *p* < 0.05 compared with the ALD group. Immunofluorescence staining was used to detect the coexpression of (**C**) α-SMA and M-CSF; (**D**) α-SMA, F4/80, and M-CSFR; and (**E**) α-SMA, F4/80, and p-MCSFR in VSMCs. Scale bars = 100 μm. *n* = 3. ALD: aldosterone stimulation; ESA: esaxerenone treatment; M-CSF: macrophage colony-stimulating factor; and M-CSFR: macrophage colony-stimulating factor receptor.

To clarify how aldosterone regulates the transformation of VSMCs into macrophage-like cells, immunohistochemical staining was used to detect the expression of macrophage colony-stimulating factor (M-CSF), M-CSF receptor (M-CSFR), and p-MCSFR in the rat aortas. The expression of these three indicators was greater in the ALD group than in the Sham group, whereas their expression was significantly lower in the ESA group than in the ALD group (Figure 6A). The results of the Western blot analysis of M-CSF expression in the aorta were consistent with the above results (Figure 6B). Immunofluorescence staining was used to detect the coexpression of α-SMA, M-CSF, F4/80, and p-MCSFR in the VSMCs, and upon aldosterone stimulation, the coexpression of α-SMA and M-CSF; F4/80, α-SMA, and M-CSF; and α-SMA, F4/80, and p-MCSFR was significantly greater than that in the CON group; these results are consistent with the changes in related indicators observed in arterial tissues (Figure 6C–E).

**Figure 7:**
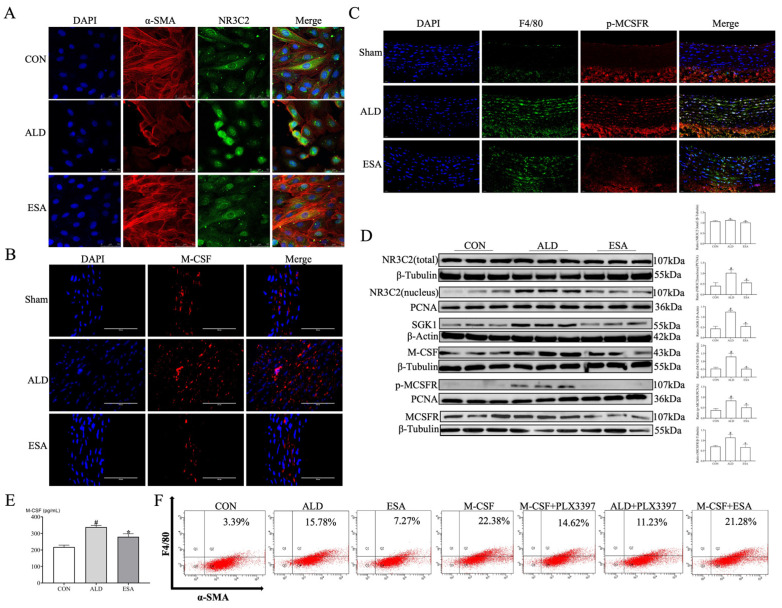
Aldosterone activates the MR/M-CSF/M-CSFR pathway. (**A**) The expression of NR3C2 in VSMC nuclei. Scale bar = 50 μm. *n* = 3. (**B**) Immunofluorescence analysis of M-CSF in the aortas of aldosterone-infused rats. Scale bars = 100 μm. *n* = 3. (**C**) The co-expression of F4/80 and p-MCSFR were detected by immunofluorescence staining; F4/80 is shown in green, p-MCSFR is shown in red, and DAPI is shown in blue. Scale bars = 20 μm. *n* = 3. (**D**) Western blot analysis of the expression of NR3C2 (total and nuclear), SGK1, M-CSF, p-MCSFR, and M-CSFR in VSMCs. *n* = 6. (**E**) The concentration of M-CSF in the VSMCs culture medium was detected via ELISA. *n* = 6. (**F**) Flow cytometry analysis of VSMCs after different treatments (ALD, ESA, M-CSF, M-CSF+PLX3397, ALD+PLX3397, and M-CSF+ESA). ^#^ *p* < 0.05 compared with CON; * *p* < 0.05 compared with ALD. ALD: aldosterone stimulation; ESA: esaxerenone treatment; SGK1: serum- and glucocorticoid-inducible kinase 1; NR3C2: nuclear receptor subfamily 3, group C, member 2; and MR: mineralocorticoid receptor.

Furthermore, immunofluorescence staining was used to detect the activation of MR in the VSMCs. MR belongs to the nuclear receptor subfamily 3, group C, member 2 (NR3C2). After aldosterone stimulation, MR translocated from the cytosol to the nucleus, indicating its activation, while the nuclear expression of MR in the ESA group was lower than that in the ALD group (Figure 7A). M-CSF expression and the coexpression of F4/80 and p-MCSFR in the aortas of the ALD group were also greater than those in the Sham group, whereas those in the ESA group were lower (Figure 7B,C). Moreover, the Western blot result for MR activation-related proteins showed that compared with those in the CON group, the nuclear expression of NR3C2 and the expression of serum- and glucocorticoid-inducible kinase 1 (SGK1), M-CSF, p-MCSFR, and M-CSFR were increased in the ALD group, whereas the expression of these proteins was decreased in the ESA group (Figure 7D). Additionally, VSMCs were cultured in vitro, and the concentration of M-CSF in the culture medium was measured. Compared with that in the CON group, the level of M-CSF in the ALD group was significantly greater, whereas the level of M-CSF in the ESA group was lower than that in the ALD group (Figure 7E). To validate the role of aldosterone and M-CSF in the transformation process, we stimulated the VSMCs with aldosterone, M-CSF, and their respective inhibitors, i.e., esaxerenone and PLX3397 (a small molecule inhibitor of M-CSFR kinase signaling), before detecting F4/80 and α-SMA expression by flow cytometry. The proportion of F4/80^+^α-SMA^+^ cells was greater in both the ALD and M-CSF groups than that in the CON group, whereas compared with those in the ALD and M-CSF groups, the proportions of double-positive cells in the ESA and M-CSF+PLX3397 groups were lower, respectively. Interestingly, the proportion of double-positive cells was also lower in the ALD+PLX3397 group than in the ALD group, whereas no significant difference in the proportion of double-positive cells was detected in the M-CSF+ESA group compared with the M-CSF group (Figure 7F). These results suggest that both M-CSF and aldosterone are involved in the VSMC-to-macrophage-like cell transformation and that, although M-CSF functions downstream of aldosterone, their effects are similar. Additionally, PLX3397 can inhibit aldosterone stimulation, whereas the inhibitory effects of esaxerenone on M-CSF are weaker; thus, aldosterone may stimulate the transformation of VSMCs into macrophage-like cells by activating the MR/M-CSF/M-CSFR pathway.

## 3. Discussion

VSMCs exhibit high plasticity, indicating that mature, differentiated VSMCs can transition between contractile and synthetic phenotypes. Under normal physiological conditions, VSMCs are fully differentiated and mature, exhibit a contractile phenotype, and show high levels of contractile proteins, including α-SMA, smooth muscle myosin heavy chain (SM-MHC), smooth muscle 22α (SM22α), and calponin [21]. However, VSMCs in damaged blood vessels undergo dedifferentiation from the contractile phenotype to the synthetic phenotype, which is characterized by increased proliferation and migration capabilities, increased production and secretion of extracellular matrix components, and decreased expression of specific contractile VSMC markers [22,23]. There are various VSMC phenotypic transformations, including macrophage-like, fibrochondrocyte-like, and fibroblast-like transformations [24,25]. VSMC phenotypic transformation is an important pathological basis for various CVDs, such as atherosclerosis, hypertension, pulmonary arterial hypertension, and aortic dissection [26,27,28]. Among these transformations, the VSMC macrophage-like transformation has received considerable attention and was confirmed in this study. We used aldosterone infusion to induce inflammatory vascular lesions in rats and found increased expression of macrophage markers and inflammatory mediators in the vascular media. Further studies revealed that these cells coexpressed markers of both smooth muscle cells and macrophages, suggesting that these macrophages may originate from the phenotypic changes in VSMCs.

As one of the main immune cells, macrophages can regulate proinflammatory cytokines through antigen presentation, polarization, and phagocytosis. Compared with macrophages derived from monocytes, which have strong phagocytic capabilities, these VSMC-derived macrophage-like cells exhibit much weaker phagocytic ability [29,30]. The SMC subtypes were detected by single-cell transcriptome and SMC-lineage tracing technology, which revealed that SMC5 had proinflammatory features characterized by phagosome involvement in function and inflammation-related signal transduction, while trajectory analysis and pseudotime calculations showed that the coexpression of SMC and macrophage lineage markers was a molecular characteristic of SMC5 [31]. Additionally, these VSMCs can promote the migration and aggregation of immune cells by secreting chemokines such as MCP-1 and intercellular cell adhesion molecule-1 (ICAM-1), leading to chronic and severe inflammatory responses in blood vessels [6,32]. This may also be one of the mechanisms by which macrophage-like VSMCs induce or participate in inflammatory vascular lesions. Macrophage can also participate in inflammatory responses through polarization. Activated macrophages are usually considered to be of the M1 or M2 type. M1 macrophages are involved in the inflammatory response by producing and secreting proinflammatory cytokines such as IL-1β, IL-6, and TNF-α, while the main functions of M2 macrophages are to inhibit inflammation, clear cellular debris and apoptotic cells, and promote tissue repair and fibrosis [33]. In this study, after aldosterone stimulation, the proportion of M1 macrophages among the macrophage-like VSMCs was greater than that of the M2 macrophages, revealing that the functions of these macrophage-like VSMCs may be more similar to those of M1 macrophages. However, the role and mechanism of VSMCs in inflammatory vascular lesions need further in-depth study.

Proinflammatory cytokines play crucial roles in the proliferation, migration, and phenotypic transformation of VSMCs. When the vascular endothelium is damaged and macrophages are activated, TNF-α is released and binds to VSMC surface receptors, stimulating the secretion of platelet-derived growth factor (PDGF), which promotes the proliferation of VSMCs. Furthermore, TNF-α increases the expression of ICAM-1 in VSMCs and enhances monocyte adhesion, leading to MAPK phosphorylation, NF-κB activation, and IL-1 and IL-8 upregulation, resulting in VSMC phenotypic transformation and vascular inflammation [34]. Here, we focused primarily on the proinflammatory effects of aldosterone. Aldosterone, a mineralocorticoid synthesized and secreted mainly by the adrenal cortex, is also the terminal effector hormone of the renin-angiotensin-aldosterone system (RAAS) activation. Aldosterone is known to participate in regulating water and sodium homeostasis in the kidney to maintain blood volume and electrolyte balance [35]. However, aldosterone is increasingly recognized as a critical factor in CVD development, which is closely related to its strong proinflammatory effects.

Aldosterone binds to MR, forming an aldosterone–MR complex and activating MR. This complex enters the nucleus and binds to aldosterone response elements in DNA, regulating the transcription and translation of its target genes, inducing the expression of the associated proteins, and thereby exerting biological effects. MR is expressed not only in the kidney and heart but also in the vasculature, such as in vascular endothelial cells and VSMCs. Numerous studies indicate that vascular MR overactivation can contribute to vascular inflammatory responses, vascular remodeling and fibrosis, and the development of atherosclerosis, making it an important mechanism in the pathogenesis of CVDs [36,37,38]. Aldosterone-activated MR regulates the expression of target genes, such as placental growth factor (PIGF), promoting the proliferation of VSMCs and the aggregation of monocytes and contributing to the development of atherosclerosis [39]. Both in vivo and in vitro studies have confirmed that aldosterone can upregulate galectin 3 (Gal-3), which is involved in vascular inflammatory responses, type I collagen deposition, vascular fibrosis, and vascular remodeling [40]. Aldosterone can also activate MR in VSMCs, thereby mediating the activation of type III sodium-dependent phosphate transporter (PIT-1), causing VSMCs to undergo osteo- and chondrogenic phenotype transformation and leading to vascular calcification [41]. This study confirmed the relationship between MR activation and VSMC transformation into macrophage-like cells. MR activation and the subsequent increase in the expression of its downstream products are involved in the formation of macrophage-like VSMCs. These findings indicate that aldosterone-induced inflammation may be a key factor in the VSMC-to-macrophage-like cell transformation.

M-CSF, a hematopoietic growth factor that not only regulates the maturation, proliferation, differentiation, and migration of macrophages but also participates in bone metabolism and inflammatory responses. In inflammatory diseases, M-CSF expression increases, which promotes the transformation of monocytes into macrophages and macrophage proliferation, maturation, and migration [42]. Studies have shown that the M-CSF content in the arterial vessels of atherosclerosis patients is increased, with oxLDL being an important factor in its synthesis and secretion. M-CSF binds to its receptor M-CSFR, triggering the dimerization, phosphorylation, and subsequent activation of M-CSFR. p-MCSFR can directly or indirectly activate the AKT and PI3K pathways to regulate macrophage survival and activate the MEK and PI3K pathways to regulate macrophage proliferation [43]. M-CSF/M-CSFR signaling is closely related to the development of various inflammatory diseases and CVDs. Research has demonstrated a strong association between circulating M-CSF levels and CVD risk [44]. During atherosclerosis development, the expression of M-CSFR in infiltrating immune cells is significantly increased, whereas treatment with the M-CSFR inhibitor GW2580 can inhibit monocyte migration and ameliorate atherosclerotic lesions [45]. Under inflammatory stimulation, the secretion of M-CSF from aortic smooth muscle cells is increased [42]. Therefore, interventions targeting M-CSFR may become a new treatment modality for various inflammatory diseases and CVDs.

However, how aldosterone-activated MR in VSMCs affects the synthesis and secretion of M-CSF is currently poorly understood. Aldosterone activates MR in VSMCs, and the gene encoding MR is located on chromosome 4q31 [46]. MR can bind many molecular chaperones, such as heat shock protein 90 (HSP90), which is essential for maintaining the conformation of MR upon ligand binding. Aldosterone binds to MR, causing the dissociation of MR from its chaperone protein, translocation to the nucleus, and induction of the transactivation and regulation of hundreds of target genes [47]. In this study, we found that the expression of M-CSF in the blood vessels of aldosterone-infused rats was increased and that the expression of M-CSFR on the surface of VSMCs that underwent macrophage-like transformation was also increased. Aldosterone induced the macrophage-like transformation of VSMCs in vitro, which was associated with increased expression of M-CSF and M-CSFR induced by MR activation. The aldosterone receptor antagonist (MRA) esaxerenone inhibited the M-CSF/M-CSFR-regulated macrophage-like transformation of VSMCs and alleviated inflammatory vascular lesions. These results demonstrate the contributions of aldosterone and M-CSF to inflammatory vascular lesions. The use of the MRAs and M-CSF inhibitor PLX3397 further clarified the upstream and downstream relationships between MR and M-CSF.

This study has several limitations. First, the detailed mechanism by which VSMCs transform into macrophage-like VSMCs still needs further exploration. Understanding how MR activates M-CSF and its receptor is crucial for preventing vascular inflammatory damage and CVD. Second, whether macrophage-like VSMCs have functions similar to those of M1 macrophages and their role in vascular injury have yet to be elucidated.

In summary, aldosterone activates MR in VSMCs, regulating the transformation of these cells into macrophage-like cells through the MR/M-CSF/M-CSFR pathway. Macrophage-like VSMCs then secrete various inflammatory mediators, further exacerbating vascular inflammation. The MRA esaxerenone can inhibit this transformation by suppressing the MR/M-CSF/M-CSFR pathway, thereby reducing inflammatory vascular lesions.

## 4. Materials and Methods

### 4.1. Animals and Study Design

Thirty male SPF Wistar rats (4~5 weeks, 165 ± 15 g) were selected for this study. The animals were maintained under controlled conditions (24 ± 2 °C, 12-h light/dark cycle) with standard food and tap water. All animal experiments in this study were approved by the Animal Experimental Ethics Committee of Hebei University of Chinese Medicine (number: DWLL202202015). Animal care followed the Animal Control Regulations of the Ministry of Health of the People’s Republic of China (document no. 55, 2001) and the Animal Health Use Committee of Hebei University of Chinese Medicine.

Following a 7-day adaptation period, the rats were randomized into 3 groups (*n* = 10 rats/group): the Sham group (Sham), the aldosterone group (ALD), and the esaxerenone group (ESA). A small suction pump (ALZET model 2006, DURECT Corporation, Cupertino, CA, USA) was implanted subcutaneously into the rats in the ALD group and the ESA group based on previous studies and the manufacturer’s guidelines for continuous infusion with aldosterone (CAS no. 52-39-1, Cayman Chemical, Ann Arbor, MI, USA) at a dosage of 600 μg/kg/d and replaced after six weeks. In accordance with the literature and the manufacturer’s directions, esaxerenone was added to the diet of the ESA group at a dosage of 1 mg/kg/day, courtesy of the Daiichi Sankyo Co., Ltd., Tokyo, Japan. Twelve weeks after the operation, the rats were euthanized and their aortas were obtained for histological analysis.

### 4.2. Blood Pressure

The systolic blood pressure (SBP) and diastolic blood pressure (DBP) were measured every week by the tail-cuff method (BP-2000, Visitech Systems, Apex, NC, USA) to observe blood pressure variations.

### 4.3. Intravascular Ultrasound Imaging of the Aorta

Ultrasound examinations were performed on the rats under inhalation anesthesia with isoflurane (1–2.5%). The thickness of the media layer in the aortic was detected and longitudinally monitored using a high-resolution ultrasound system for small animals (Vevo^®^ 2100 Imaging System, FUJIFILM VisualSonics Inc., Toronto, Canada; EZ-SA800 Single Animal System, E-Z Systems Inc., Commonwealth of Pennsylvania, St. Harrisburg, PA, USA).

### 4.4. In Vitro Cell Culture Assays

VSMCs were obtained from the Bena culture collection (Xinyang, Henan Province, China). The cells were cultured in a medium consisting of 90% DMEM-H and 10% FBS (SA211.02, CellMax, Lanzhou, China) at 37 °C in 5% CO_2_ incubators. When the cells reached 60–80% confluence, the cells were randomly assigned to three groups: the CON group, the ALD group, and the ESA group. The dosages of ALD and ESA were as previously described [48]. The VSMCs were treated with M-CSF (MCE, Shanghai, China, cat. no. Hy-p7085) at a dose of 10^−7^ mol/L in the subsequent experiments with or without the M-CSF receptor inhibitor PLX3397 (MCE, Shanghai, China, cat. no. Hy-16749) at a dose of 10^−7^ mol/L. After 12 h, the cells were collected for further experiments.

### 4.5. Histopathological and Immunohistochemical Analyses

The aortas were embedded and sectioned into 5 μm slices for H&E and immunohistochemical staining. Immunohistochemical staining was performed to analyze F4/80 (1:100, Servicebio, Wuhan, China, cat. no.GB113373), CD68 (1:100, Abcam, Cambridge, UK, cat. no. ab125212), MCP-1 (1:100, Zenbio, NC, USA, cat. no. 507277), IL-1β (1:100, Zenbio, NC, USA, cat. no. 516288), TNF-α (1:100, Servicebio, Wuhan, China, cat. no. GB11188), M-CSF (1:100, Abcam, Cambridge, UK, cat. no. ab233387), and M-CSFR (1:100, Abcam, Cambridge, UK, cat. no. ab183316) levels. A Leica BX53 optical microscope (Leica, Wetzlar, Germany) was used to observe and photograph the samples.

### 4.6. Immunofluorescence Staining

The rat aortas were embedded in OCT compound (Sakura, Torrance, CA, USA); then, 6 μm thick frozen sections were prepared for subsequent immunofluorescence staining. The primary antibodies against M-CSF (1:100, Abcam, Cambridge, UK, cat. no. AB233387), F4/80 (1:200, Servicebio, Wuhan, China, cat. no. GB113373), and p-MCSFR (1:500, Affinity, Nanjing, China, cat. no. AF4394) were added and incubated at 4 °C for 24 h. Then, the secondary antibody Alexa Fluor^®^ 488/555 (1:200, Abcam, Cambridge Science Park, Cambridge, UK) was added, and the mixture was incubated at 37 °C for 1 h.

### 4.7. Cellular Immunofluorescence Analysis

After fixation, the cells were subjected to immunofluorescence analysis, and primary antibodies against F4/80 (1:200, Servicebio, Wuhan, China, cat. no. GB113373), M-CSF (1:100, Abcam, Cambridge, UK, cat. no. ab233387), M-CSFR (1:100, Abcam, Cambridge, UK, cat. no. ab183316), p-MCSFR (1:500, Affinity, Nanjing, China, cat. no. AF4394), and α-SMA (1:100, Abcam, Cambridge, UK, cat. no. ab202509) were added and incubated at 4 °C for 24 h. Then, the secondary antibody conjugated to Alexa Fluor 488 (1:200, Abcam, Cambridge, UK) was added, and the mixture was incubated for 1 h at 37 °C. The nuclei were stained with DAPI (C0065, Solarbio, Beijng, China) at room temperature for 10 min. Observation and analysis were performed using a confocal microscope (CTS SP8, Leica, Germany) and an EVOS^®^ FLAuto workstation (Thermo Fisher, Waltham, MA, USA).

### 4.8. Protein Extraction and Western Blot Analysis

To extract protein or nucleoproteins, the aortas and cells were lysed in cold RIPA buffer (BB-3201, Bestbio, Shanghai, China). SDS-PAGE was used to separate the samples, and a PVDF membrane (R1NB77899, Merck Millipore Ltd., Tullagreen, Carrigtwohill, Co., Cork, Ireland) was used for transfer. The samples were incubated with primary antibodies against NR3C2 (1:500, Proteintech, Chicago, IL, USA, cat. no. 21854-1-AP), M-CSF (1:500, MCE, Shanghai, China, cat. no. HY-P7085), M-CSFR (1:500, Affinity, Nanjing, China, cat. no. AF0080), p-MCSFR (1:500, Affinity, Nanjing, China, cat. no. AF4394), IL-1β (1:1000, Zenbio, NC, USA, cat. no. 516288), TNF-α (1:100, Servicebio, Wuhan, China, cat. no. GB11188), MCP-1 (1:500, Affinity, Nanjing, China, cat. no. DF7577), SGK1 (1:200, Affinity, Nanjing, China, cat. no. DF6188), glyceraldehyde-3-phosphate dehydrogenase (GAPDH; 1:1000, Proteintech, Chicago, USA, cat. no. 60004-1-Ig), β-actin (1:1000, Proteintech, Chicago, USA, cat. no. 66009-1-Ig), β-Tubulin (1:1000, Affinity, Nanjing, China, cat. no. DF7967), and PCNA (1:5000, Proteintech, Chicago, USA, cat. no. 60097-1-Ig) at 4 °C overnight. A secondary antibody (1:5000, IRDye 680RD/IRDye 800CW, LI-COR Biosciences, Lincoln, NE, USA) was incubated with the membrane for 1 h at room temperature the following day. Protein bands were detected using e-BLOT (Shanghai e-BLOT Photoelectric Technology Co., Ltd. Shanghai, China), and protein expression was quantified using ImageJ software (version 1.8.0).

### 4.9. Enzyme-Linked Immunosorbent Assays (ELISAs)

M-CSF concentrations in the culture supernatant were determined using an M-CSF ELISA kit (rat M-CSF ELISA kit, Abcam, Cambridge, UK, cat. no. AB253214). The samples or reference standards (100 μL) were added to the microwell plates and incubated at room temperature for 60 min. After washing, horseradish peroxidase (HRP)-conjugated polyclonal secondary antibodies were added (100 μL/well) and incubated in the dark for 30 min. After washing, 100 μL of developer solution (3,3′,5,5′-tetramethylbenzidine) was added, and the mixture was incubated in the dark for 20 min, after which 50 μL of termination solution was added. The results were quantified after the optical density was measured.

### 4.10. Flow Cytometry

The cells were harvested and rinsed with cold PBS after 12 h of treatment and incubated with anti-F4/80 (1 μL/test, Thermo Fisher, Massachusetts, USA, cat. no. 11-4801-85), anti-iNOS (0.5 μL/test, Thermo Fisher, Massachusetts, USA, cat. no. 25-5920-80), anti-CD206 (0.5 μL/test, BioLegend, San Diego, CA, USA, cat. no. 141705), and anti-α-SMA (0.5 μL/test, Abcam, Cambridge, UK, cat. no. ab202296) antibodies for 1 h, followed by incubation with the corresponding secondary antibodies (Alexa Fluor 647, 1:500, Abcam, Cambridge, UK, cat. no. Gr3176223-1) in the dark. Unstained cells were used as negative controls. A BD FACS ARIA II flow cytometer (BD Biosciences, Franklin Lake, NJ, USA) was used to analyze the cells. FSC/SSC gating was used to select live singlet cells, and the data were analyzed with FlowJo 10 software.

### 4.11. Statistical Analysis

The measured data are expressed as means ± standard deviations. Statistical analysis between groups was conducted using Student’s *t* test or one-way ANOVA, followed by Tukey’s post hoc test for multiple groups and the chi-square test. Statistical significance was indicated by a *p* value < 0.05.

## Data Availability

The data supporting the findings of this study are available from the corresponding author upon reasonable request.

## Supplementary Materials

The following supporting information can be downloaded at https://www.mdpi.com/article/10.3390/ijms26073345/s1.

## Abbreviations

The following abbreviations are used in this manuscript:

VSMCs	vascular smooth muscle cells
M-CSF	macrophage colony-stimulating factor
MR	mineralocorticoid receptor
RAAS	renin–angiotensin–aldosterone system
TNF-α	tumor necrosis factor-α
MCP-1	monocyte chemoattractant protein-1
IL-1β	interleukin-1β
M-CSFR	macrophage colony-stimulating factor receptor
CVD	cardiovascular disease
CHD	coronary heart disease
MRs	mineralocorticoid receptors
MRAs	mineralocorticoid receptor antagonists
Sham	Sham operation group
ALD	aldosterone group
ESA	esaxerenone group
DMEM	Dulbecco’s modified Eagle’s medium
FBS	fetal bovine serum
DMSO	dimethyl sulfoxide
PFA	paraformaldehyde
DAPI	4′,6-diamino-2-phenylindole
RIPA	radioimmunoprecipitation assay
PVDF	polyvinylidene fluoride
HRP	horseradish peroxidase
PBS	phosphate-buffered saline
ANOVA	analysis of variance
α-SMA	α-smooth muscle actin
SM-MHC	smooth muscle myosin heavy chain
SM22α	smooth muscle 22α
ICAM-1	intercellular cell adhesion molecule-1
TNF	tumor necrosis factor
PDGF	platelet-derived growth factor
PIGF	placental growth factor
Gal-3	galectin 3
PIT-1	sodium-dependent phosphate transporter
NR3C2	nuclear receptor subfamily 3, group C, member 2
HSP90	heat shock protein 90
MRA	aldosterone receptor antagonist
SGK1	serum- and glucocorticoid-inducible kinase 1
