# Supplementary material for: Aldosterone-Induced Transformation of Vascular Smooth Muscle Cells into Macrophage-like Cells Participates in Inflammatory Vascular Lesions
Source: Int J Mol Sci. 2025 Apr 3;26(7):3345. doi: 10.3390/ijms26073345 (PMC11989480; doi:10.3390/ijms26073345)
Supplement: Supplementary file 1 [file ijms-26-03345-s001.zip › ijms-3449782-supplementary.pptx]

## Slide 1
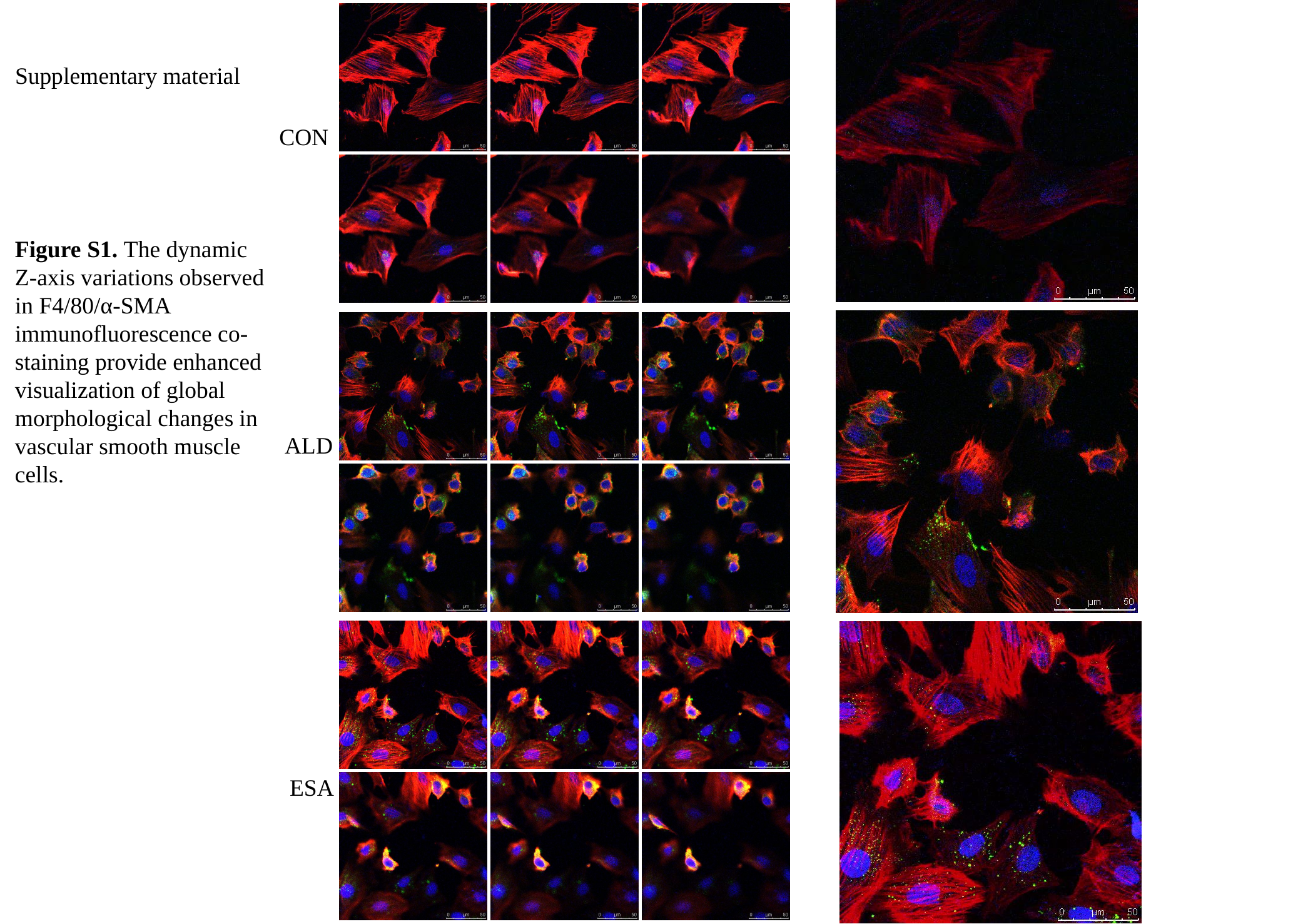

CON
Supplementary material
Figure S1. The dynamic Z-axis variations observed in F4/80/α-SMA immunofluorescence co-staining provide enhanced visualization of global morphological changes in vascular smooth muscle cells.
ALD
ESA

## Slide 2
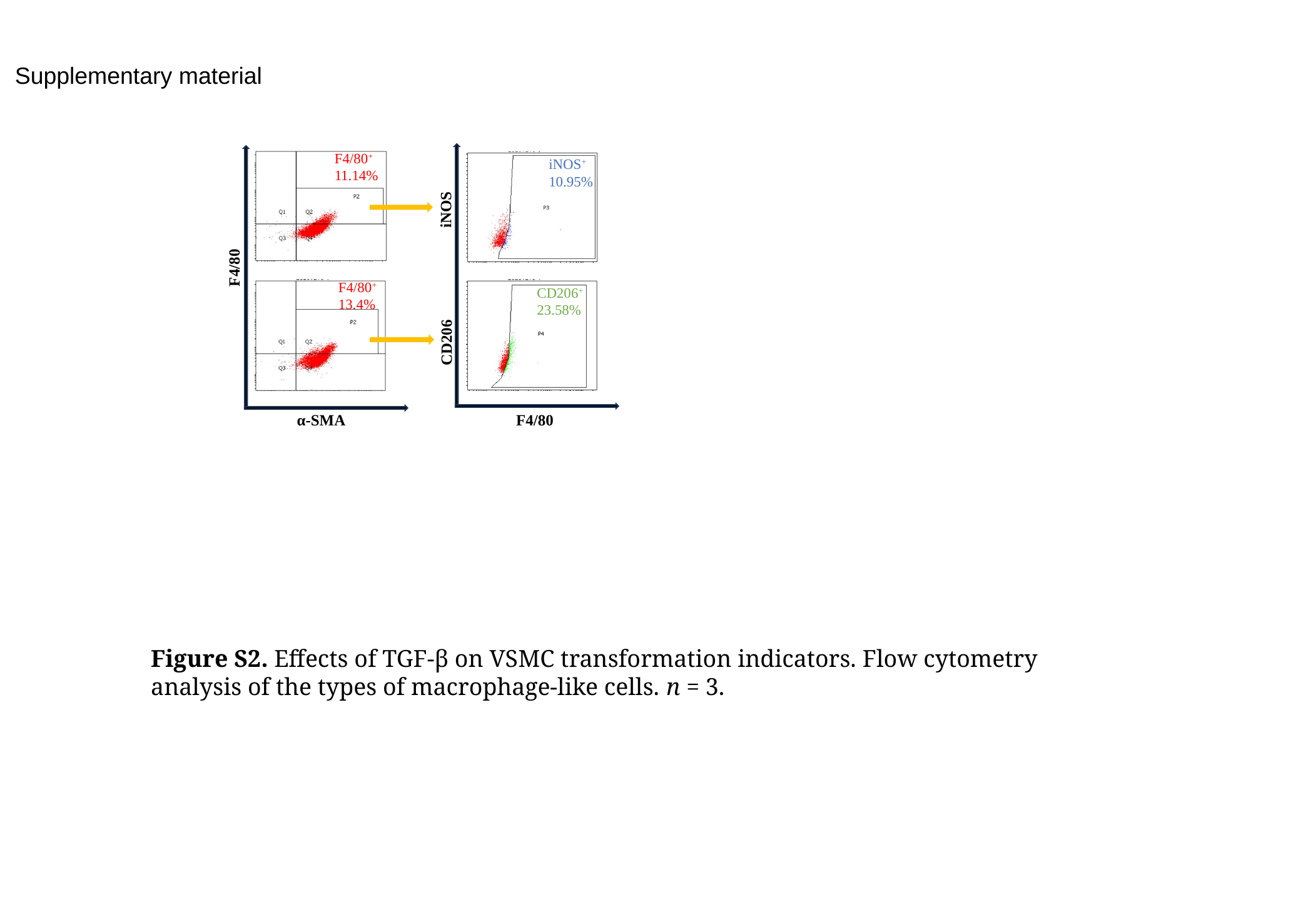

Supplementary material
iNOS
F4/80
CD206
α-SMA
F4/80
F4/80+
11.14%
iNOS+
10.95%
F4/80+
13.4%
CD206+
23.58%
Figure S2. Effects of TGF-β on VSMC transformation indicators. Flow cytometry analysis of the types of macrophage-like cells. n = 3.
